# Communicative mentalization is limited in nonhuman great apes

**DOI:** 10.1073/pnas.2503448122

**Published:** 2025-05-16

**Authors:** Tibor Tauzin

**Affiliations:** ^a^Department of Linguistics, University of Vienna, Vienna 1090, Austria; ^b^Vienna Cognitive Science Hub, University of Vienna, Vienna 1090, Austria

The evolutionary origins of humans’ mentalization skills have been investigated since the seminal paper by Premack and Woodruff (1). In his famous response to this paper, Dennett (2) argued that empirical tasks testing mentalization must be novel for the participants. Thus, they should not rely on the previous experiences of the subjects, because in such contexts, their behavioral responses can reflect simpler, reward-based learning mechanisms.

Townrow and Krupenye (3) claim that bonobos can mentalize in communicative interactions because they point more frequently when they inform an ignorant as opposed to a knowledgeable human about the location of a food item. However, their study design does not meet the novelty requirement; therefore, it is open to alternative interpretations. In the familiarization phase and repeated familiarization trials, apes were taught that an experimenter—who saw the hiding of the food—would reveal and provide it for them, even if the apes did not point. As expected, apes produced few points in the familiarization trials, presumably because it was not necessary to obtain a reward. The knowledge test trials were virtually the same as the familiarization trials, so the apes might continue what they had learned during familiarization. Indeed, they pointed infrequently in the knowledge condition. In contrast, during the ignorance test trials, the hiding of the food was occluded from the experimenter by a screen, and the apes produced more pointing gestures. These results may suggest that bonobos attributed ignorance to the experimenter regarding the food’s location. However, it is also possible that they pointed less in the knowledge than in the ignorance condition because they had already learned that it was not necessary to point to receive food. In contrast, in the ignorance condition, apes might point more often, because they were rewarded only in one-third of the trials if they did not point. Or perhaps, they pointed more because they had learned in previous interactions with humans that pointing increases their chances to receive a reward—unless they had been taught otherwise. These or similar reward-based learning processes, whether alone or combined, could account for the apes’ behavior in the Townrow and Krupenye (3) study.

This is why the novelty of tasks is crucial for testing mentalization skills. In line with this idea, we designed a paradigm in which apes (4) or human infants (5) had to modify their pointing gestures spontaneously and without prior practice to indicate the location of a relevant object. We found that both apes and humans were able to modify their points; however, only human infants used these deictic gestures to provide their ignorant addressees (or those with a false belief) with more relevant information. Since previous experience could not have influenced our participants’ performance, and sample sizes were considerably larger than in the Townrow and Krupenye (3) study, this suggests that communicative mentalization might be human-specific—an ability that humans use spontaneously, in novel contexts, from early on with their conspecifics. This indicates that it can play a central role in human interactions, but not in those of great apes.
